# Treating chronic spontaneous urticaria using a brief ‘whole person’ treatment approach: a proof-of-concept study

**DOI:** 10.1186/s13601-015-0082-7

**Published:** 2015-12-02

**Authors:** Karen Lindsay, Josie Goulding, Margot Solomon, Brian Broom

**Affiliations:** Department of Immunology, Auckland City Hospital, Park Rd, Grafton, Auckland, 1023 New Zealand; Department of Psychotherapy and Counselling, Auckland University of Technology, Akoranga Rd Campus, Auckland, New Zealand

**Keywords:** Chronic urticaria, Efficacy, Treatment, Whole person treatment approach, Psychotherapy

## Abstract

**Background:**

Chronic spontaneous urticaria (CSU) poses problems with respect to high prevalence, reduced quality of life, lack of long term efficacy, and expense of current treatments for severe intractable symptoms. There have been many reports suggesting ‘stress’ factors may be implicated, but there are no studies that explore the efficacy of treatments including a psychological perspective. A whole person treatment approach (WPTA), which addresses psychological factors has been used, with effect, for 6 years in the Auckland City Hospital Immunology Department.

**Findings:**

In a pilot study to demonstrate feasibility of recruitment and treatment of CSU patients in a time-limited, whole person treatment approach, within a conventional immunology department, four patients (three CSU and one idiopathic angioedema) were recruited into a brief WPTA course based in non-dualistic concepts of mind and body connectedness, and utilising psychotherapy-derived listening skills for up to 10 h long sessions, once per week. Treatment efficacy rating, using Urticaria Activity Score and the Urticaria Severity Score, and reduction of drug usage, showed patients experienced long term resolution of urticaria and cessation of hospitalisation for angioedema and came off regular antihistamine medication.

**Conclusions:**

A clinician treating chronic spontaneous urticaria in an Immunology department, using a whole person treatment paradigm, can safely explore unique meanings and emotional states, in a process acceptable to patients, resulting in a significant clinical benefit for symptoms. A much larger study comparing the outcome of WPTA versus standard treatment alone is warranted.

**Electronic supplementary material:**

The online version of this article (doi:10.1186/s13601-015-0082-7) contains supplementary material, which is available to authorized users.

## Background

Chronic spontaneous urticaria (CSU) will affect 1–5 % of the population during their lifetime [1]. This condition constitutes approximately 25 % of the referrals to the Department of Immunology at Auckland City Hospital. International drug treatment guidelines for CSU management target histamine receptors and immune mechanisms, but CSU is frequently unresponsive to antihistamine and immunosuppressive medications [2], and the impact of CSU on quality of life is often underestimated by physicians [3]. New biologic drugs targeting IgE antibodies such as omalizumab can induce but not maintain long-term remission [4]. The medical literature suggests a role for stress [5–7] and emotional factors in CSU [8]. Since 2008, in our department, many CSU patients have been treated with an individualised, non-dualistic whole person treatment approach (WPTA) (see Additional file 1: Appendix 1) [9–13]. This small pilot study was conducted to demonstrate grounds, acceptability, feasibility, and potential usefulness of a whole person approach, and to develop guidelines for a larger formal outcome study.

## Methods

The WPTA [9, 11] actively addresses the impact on the body of all kinds of life events and relational dynamics. The WPTA clinician sees the patient’s wheals or angioedema as potentially meaningful in the context of these events, and focuses upon flare-ups of CSU symptoms to access the links between mind and body. The patient and the clinician collaborate in making these connections with the expressed intention of reducing the symptoms over time. Participants were interviewed by KL, MS and JG throughout the study both by email and using face to face interviews, and the detailed analytic findings using qualitative methodologies of Grounded Theory and Interpretive Phenomenological Analysis are to be reported elsewhere.

Clinical data such as age, gender, medications, and duration of symptoms were obtained from the hospital notes. Two disease activity measures for each patient were recorded before the study at the time of consenting, and after the WPTA was completed:

1. The Urticaria Activity Score (UAS) [14]. Patients were asked to score once daily for 7 days both the number of wheals and severity of pruritus, in a range 0–3, with a combined maximum possible score of 42. Forms were then returned to the department by post or self-delivered.
2. The Urticaria Severity Score (USS) [15]. This is based on 12 questions ‘designed to measure distinct aspects of QOL impairment, the degree and duration of pruritus and/or swelling, distribution of body area with hives and/or itch, and amount of medication required to control symptoms’. It assesses severity of urticaria symptoms, and treatment required, over the previous 7 days, with a maximum score of 93 and a minimum of 0.

The study proposal was reviewed by the New Zealand Northern Y regional Ethics committee (NTY/10/05/047). In an open recruitment, patients were invited to take part by senior staff responsible for their care within the Immunology Department, a tertiary specialist service. They were given a one page patient information sheet which described the WPTA, and then contacted by the lead researcher (KL) and again invited to take part.

## Recruitment

Fifteen patients were referred and screened for entry. The study comprised the first four patients (hereafter numbered 1–4) who were both eligible and accepting of the research process. The study group comprised three females and one male, mean age 55.2. There were five more people eligible and willing in principle, but who could not enter because the prospect of 10 weekly visits to the hospital interfered unacceptably with work. Four patients rejected the WPTA as described to them by KL. The first four patients who agreed to participate became the subjects for the study. All four were naive to the WPTA at recruitment, and were told that the study involved an approach that ‘treats the mind and the body as one entity rather than as two unconnected or separate things’ and would involve up to ten sessions lasting 30–60 min, with a clinician who is both an immunologist and a trained psychotherapist (Patient Information Sheet).

Three patients (all with typical CSU of unknown aetiology) underwent ten sessions of WPTA with BB (immunologist/psychotherapist). One person (patient 4) had seven sessions. Patient 4 was originally accepted on the referral basis that he had CSU with severe angioedema, requiring repeated hospitalisations with life threatening angioedema, but turned out to have no symptoms of wheals or hives.

## Findings

The three CSU patients had persistent very frequent urticarial symptoms and were regarded by referrers as not responding to standard treatment. They were taking maximal doses of antihistamines currently available in New Zealand; cetirizine or loratadine up to 40 mg daily, with additional fexofenadine up to 180 mg daily, without relief of symptoms. No patient had ever been trialled on disease-modifying agents. The fourth patient had declined antihistamine medications as they were ineffective in preventing recurrent angioedema. Table 1 has patients’ ages, disease duration, and scores before and after the WPTA intervention. Three of four had improved UAS [16] and USS scores [17], with almost no disease activity at the end of the study, and reduced drug use to less than monthly antihistamines only, an effect which was sustained in all three at telephone follow up at 2 years post-WPTA. The fourth patient experienced reductions of serious hospitalisation for angioedema involving intubation and stays on the intensive care unit. Most patients recruited saw the WPTA as a psychotherapy/counselling-like intervention. Patients did experience coincident flares of urticaria and emotion, of which they became increasingly aware during the therapeutic sessions, or shortly afterwards. Symptom increases were treated as meaningful and were used to good effect by the clinician to make links between experience and urticaria, affect and emotion, and formed part of the therapeutic intervention.

**Table 1:** Urticaria Activity Scores (UAS) and Urticaria Severity Scores (USS) before and after whole person treatment approach sessions

Patient no. and sex	Disease duration at start of study	UAS (maximum 42)	Post-WPTA	USS (maximum score 93)	Post-WPTA
1. Female	23 months	20	0	37	0
2. Female	16 years	19	5	11	0
3. Female	6 years	12	4	11	0
4. Male	12 months	3	0	0	0

In retrospect, it was evident that during pre-referral management all four study subjects had had ‘stress’ identified as a trigger to their symptoms, and that this along with difficulty in biomedical management had in part prompted their referral to the study. Patient 4 did not score highly in the CSU-focussed questionnaires because he suffered predominantly from intermittent (but severe life-threatening) angioedema as part of his CSU.

## Discussion

The standard biomedical approach is dualistic, that is, it more or less assumes that mind and body are separate, and does not value or emphasise the importance of emotional factors to the physical manifestations of disease, and therefore they are rarely attended to. This pattern is reflected in the general approach to CSU, though one other centre has utilised both a biomedical and psychological approach [18].

This proof-of-concept study recruited four patients prospectively to a 10 week, non-dualistic, psychologically-oriented, mind AND body intervention (WPTA), with the clinician being an immunologist who is also a psychotherapist. It is the first study to show an intervention which results in a resolution of CSU in a group of treatment-resistant patients. Whilst it supports our experience of using the WPTA routinely and successfully in the Clinical Immunology Department at Auckland City Hospital, it primarily establishes a precedent for a larger comparative outcome study comparing the WPTA (in conjunction with standard drug therapy if needed) versus standard drug therapy alone.

A small number were recruited to this study, the first four available being taken. There were more who were unable (5, because of time constraints) or reluctant (4, rejected the model). The main patient objection was the requirement to attend weekly for 10 weeks. It needs to be emphasised that in the ‘real world’ of our clinic 10 weeks of treatment is *not* a standard requirement. Many patients actually do well within their first few routine scheduled clinic appointments with clinicians taking a WPTA approach.

All four patients, prior to being involved in the study, had at some level identified stress as being important in causing their urticaria, which could suggest a selection bias towards patients willing to consider this type of intervention. Most patients recruited saw the WPTA as a psychotherapy/counselling-like intervention. Their long experience of lack of success with other measures may have contributed to their adherence and attendance at the sessions. Those patients who self-identified stress as an exacerbating factor may have been more likely to be invited (by the referring clinician), to take part and to complete the study, and to experience benefit from this type of approach. Patients do experience coincident flares of urticaria and emotion, of which they become increasingly aware during therapeutic sessions, or shortly afterwards. From a theoretical perspective, such co-occurrence (whilst keeping in mind other physical factors) supports the notion that emotional factors are important in the patient with urticaria [5, 6, 8] and that sometimes symptoms may be mobilised by the therapeutic process. In our experience and practice, this is rarely a problem, but needs to be taken into account in future studies, and strategies put in place to manage it.

## Conclusion

This study provides proof-of-concept evidence that a whole person approach is both feasible and acceptable in a biomedical clinical setting and potentially therapeutically powerful for at least some patients, when used in tandem with standard care. The results of this study, while limited to a few patients, support the literature reporting the importance of emotional factors in CSU. A much larger outcome study of the whole person approach in direct comparison with the use of standard drug therapy is warranted.

## Additional file

10.1186/s13601-015-0082-7 The whole person treatment approach (WPTA) and its implementation in a hospital setting by physicians.
