# Supplementary material for: Treating chronic spontaneous urticaria using a brief ‘whole person’ treatment approach: a proof-of-concept study
Source: Clin Transl Allergy. 2015 Dec 2;5:40. doi: 10.1186/s13601-015-0082-7 (PMC4667404; doi:10.1186/s13601-015-0082-7)
Supplement: Supplementary file 1 — 10.1186/s13601-015-0082-7 The whole person treatment approach (WPTA) and its implementation in a hospital setting by physicians. [file 13601_2015_82_MOESM1_ESM.docx]

**Appendix 1:** The whole person treatment approach (WPTA) and its implementation in a hospital setting by physicians.

The WPTA [[1](#_ENREF_1), [2](#_ENREF_2)] actively addresses the impact on the body of all kinds of life events and relational dynamics. The patient’s urticaria or angioedema is seen as emerging in relation to these factors, and the WPTA focuses upon flare ups of CSU symptoms to access the links between mind and body. The patient and the clinician collaborate in making these connections with the expressed intention of reducing the symptoms over time [[2](#_ENREF_2), [3](#_ENREF_3)].

The first requirement of the WPTA is that the clinician needs to have a non-dualistic model of personhood[[4](#_ENREF_4)]. The second requirement is the willingness and skill to ‘be with’ and educate patients into a more whole person view of their condition, a view that honours the obvious physical reality of the condition, but also allows the psycho-social factors to be rendered visible and resolved in some way.

In the clinical consultation, the WPTA requires the clinician to help the patient to recognise and express any life story experience relevant to the physical disorder. This may simply require a capacity for warm, active relating to the patient, and some skills of listening and reflecting, but may at times need more sophisticated skills of identifying emotional and relational themes. It is crucially important to emphasise that the WPTA is frequently and simply just brief education of the patient as to the possible connections of their emotions and ‘story’ with their disorder [[5](#_ENREF_5)]. But, for some, it becomes a few sessions with a listening physician, or a longer series of sessions with a psychotherapist. Participants in this study were told that the WPTA involves an approach that ‘treats the mind and the body as one entity rather than as two unconnected or separate things’ (Patient Information Sheet), and this is also indicated in normal clinical work with all patients.

The actual interventions used include psycho-education (particularly around mind and body connections), facilitating emotional expression in the session, active listening, empathetic connection, making links, emotional ‘holding’, and helping find language for what is happening inside and outside of the clinical room. Metaphor is frequently used for finding suitable language. Because much of the psychosocial material involved has to do with human relationships, a key to clinical effectiveness is the capacity of the clinician to engage openly and naturally, in the consultation, with what is happening *between* the clinician and patient. For example, the very circumstance of raising the question of stress and emotional factors in the clinic tests the capacity of both the patient and the clinician to engage in a delicate and potentially threatening encounter. Done well this can lead very rapidly to new dimensions of trust and a sense of being understood, encourage hope, mobilise patient motivation to deal with relevant life circumstances, and model for the patient what *can* happen, without disaster, between two people, emboldening the patient to address the relevant problematic relations outside the clinic.

The standard biomedical approach is dualistic; that is, it more or less assumes that mind and body are separate, and does not value or emphasise the importance of emotional factors to the physical manifestations of disease, and therefore they are rarely attended to. This pattern is reflected in the general approach to chronic spontaneous urticaria. As already outlined, there is some indication that psychological factors may be important in CSU [[6-10](#_ENREF_6)], though little attention is paid to these because of the general dominance of the dualistic biomedical model of disease. Moreover there are no previous studies of the feasibility of researching the treatment of CSU from a non-dualistic, whole person or psychological perspective. In our experience, whilst patients may be aware of extraordinary events that happen to them around the onset of urticaria, the links have not been highlighted or supported by clinicians. The dualistic approach compounds a patient’s reluctance to make connections between emotion and urticaria, and frequently steers him/her to unhelpful models of their disease. Patients with intractable CSU may be forced to manage on the basis of these models, sometimes for years. Failed attempts at behavioural change to control their symptoms, based on these models, create further distress, and may contribute to the chronicity of CSU in some patients. For example, elimination diets may cause severe social restriction, fear of exposure to supposed triggers, frustration, food aversion, family disruption, and even malnutrition where dietary measures are taken too far. All this has a tendency to increase stress and may make the CSU worse.

The WPTA treatment model is relevant to primary care physicians, dermatologists and immunologists. The study showed that the WPTA is acceptable to patients familiar with a medical approach, although initially participants may be anxious about being labelled as ‘psychosomatic’, and look at the WPTA as counselling or psychotherapy, and are wary of exposure and potential stigmatisation, the handling of which might explain the failure to engage reported elsewhere [[11](#_ENREF_11)]. The WPTA involves empathic, sensitive, non-stigmatising, non-psychiatrising education regarding being a ‘whole person’, and a strong view that minds and bodies are not separate. Our experience is that a significant number of CSU patients come into remission with deployment of the WPTA by doctors who are not psychotherapists. An unpublished audit (BB) of a serial group of 22 patients with CSU drawn from a coding database indicates this. All drawn patients had been coded as CSU with identifiable stress or ‘whole person’ factors, ascertained in the initial immunological consultation. The one other selection requirement was that they had not been seen for at least 6 months. In the audit they were asked how they were in respect of CSU symptoms, and what was their interpretation of their progress? The audit revealed that 13/22 patients got better once connections were made between problematic feelings and their symptoms, and these patients required only the initial allergy/WPTA assessment and in some cases one further follow-up session for this to be achieved. 9/22 patients went into a series of WPTA/counselling sessions and 7/9 got better during that process. Both groups saw their improvement in terms such as: ‘once I knew that my hives were about this (e.g. relational or life issue) I knew what to do’. 2/22 patients were still struggling, and were still in treatment. The point is that the WPTA must be geared to the individual needs of each patient, many of whom require very little more than education about the connections between emotional experience and urticarial exacerbation.

Those patients who self-identified stress as an exacerbating factor may be more likely to experience benefit from this type of approach. Nevertheless, the experience in our department is that initial reluctance frequently converts to willingness, once genuine links of CSU to emotions are made in an empathic, non-stigmatising, and non-psychiatrising environment.

The study was structured around an arbitrary maximum of ten sessions for each patient. Our experience suggests many patients can be helped with less, but some patients need more. Ten sessions seemed adequate to cover this variability for the purposes of the study and were manageable within our department’s resources. Any future outcome study would preferably tailor the amount of therapy to the minimum required to relieve symptoms in a sustainable way both for the healthcare provider and the patient. In our department, the staff are increasingly experienced and effective in providing a minimal-type of WPTA to CSU patients. But there are some patients who have severe psychological issues, and in whom the urticaria remains intractable. They may receive more aggressive drug therapies but will also be referred, where possible, for professional psychotherapy treatment with therapists who have a strong non-dualistic, whole person conceptual framework.

1. Broom BC e. Transforming clinical practice using a mindbody approach. A radical integration. . London: Karnac Books Ltd; 2013.

2. Broom BC. Somatic Illness and the Patient’s Other Story. A Practical Integrative Mind/Body Approach to Disease for Doctors and Psychotherapists. . London: Free Association Books; 1997.

3. M. S. Object Relations Brief Therapy: The Therapeutic Relationship in Short-Term Work. Northvale, NJ: Jason Aronson; 1996.

4. BC. B. Meaning-full disease: How personal experience and meanings initiate and maintain physical illness. . London Karnac Books; 2007.

5. Broom BC. A reappraisal of the role of 'mindbody' factors in chronic urticaria. Postgraduate medical journal. 2010;86(1016):365-70. doi:10.1136/pgmj.2009.096446.

6. Staubach P, Dechene M, Metz M, Magerl M, Siebenhaar F, Weller K et al. High prevalence of mental disorders and emotional distress in patients with chronic spontaneous urticaria. Acta dermato-venereologica. 2011;91(5):557-61. doi:10.2340/00015555-1109.

7. Staubach P, Eckhardt-Henn A, Dechene M, Vonend A, Metz M, Magerl M et al. Quality of life in patients with chronic urticaria is differentially impaired and determined by psychiatric comorbidity. The British journal of dermatology. 2006;154(2):294-8. doi:10.1111/j.1365-2133.2005.06976.x.

8. Weller K, Koti I, Makris M, Maurer M. Anxiety and depression seem less common in patients with autoreactive chronic spontaneous urticaria. Clinical and experimental dermatology. 2013;38(8):870-3. doi:10.1111/ced.12190.

9. Wright RJ, Cohen RT, Cohen S. The impact of stress on the development and expression of atopy. Current opinion in allergy and clinical immunology. 2005;5(1):23-9.

10. Metz M, Krull C, Hawro T, Saluja R, Groffik A, Stanger C et al. Substance P is upregulated in the serum of patients with chronic spontaneous urticaria. The Journal of investigative dermatology. 2014;134(11):2833-6. doi:10.1038/jid.2014.226.

11. Berrino AM, Voltolini S, Fiaschi D, Pellegrini S, Bignardi D, Minale P et al. Chronic urticaria: importance of a medical-psychologic approach. Eur Ann Allergy Clin Immunol. 2006;38(5):149-52.
